# Is there truth in fiction? Lessons from readers’ responses to dementia fiction

**DOI:** 10.1136/medhum-2024-012976

**Published:** 2024-12-04

**Authors:** Jane Lugea, Carolina Fernandez-Quintanilla, Gemma Carney, Paula Devine

**Affiliations:** 1 Queen's University Belfast Faculty of Arts Humanities and Social Sciences, Belfast, UK; 2 Department of English and German Philology, University of Granada, Granada, Andalucía, Spain

**Keywords:** dementia, Literature, Linguistics, metaphor, literature and medicine

## Abstract

This paper addresses the question ‘is there truth in fiction?’, by synthesising a range of disciplinary approaches to the issue, as well as drawing on empirical research carried out with readers of fiction about dementia (hereafter, dementia fiction). We argue that fiction—perhaps because of its fictional status and apparatus—invites readers to consider its truth value, to explore the possibilities of human experience and interrogate issues relative to their subjective experience, community or society. The findings have significant implications for the Medical Humanities’ use of fictional texts to explore lived medical conditions and experiences, as well as claims made about the potential for fiction to affect real-world understandings, awareness and empathy around the conditions depicted. We show that the techniques used in fictional language may be artifice, but they simulate a truth that corresponds with reality.

## Introduction

### Contextual remarks

 This paper addresses the question ‘is there truth in fiction?’, by synthesising a range of disciplinary approaches to the issue, as well as drawing on empirical research carried out with readers of fiction about dementia (hereafter, dementia fiction). The question is increasingly important as new media and technological advancements (virtual reality and gaming) have important implications for the status of ‘fiction’, its relationship to ‘reality’ and its truth value.^1^ Focussing on narrative fiction, we demonstrate its value in understanding dementia, a social and ‘public health priority’ (WHO 2017). We address the question by applying it to a more specific problem: to what extent have authors captured the truth of dementia in fictional language? This question is answered through reader response research carried out with people living with dementia, with caring experience, students training as social workers, and members of the public with little or no direct experience of dementia.

The research underpinning this paper was carried out by an interdisciplinary group of researchers with backgrounds in literary linguistics and cultural gerontology. We devised an experimental method to explore the potential for fictional characters with dementia to evoke understanding and empathetic responses in readers, a possibility proposed by several scholars (eg, Zimmermann 2010). The research took place over several years and has led to publications on the corpus of dementia fiction (Lugea 2022a), on how carers versus people with dementia respond to the fiction (Carney *et al* 2023) and outlining the innovative research methods (Devine *et al*, forthcoming). However, in the process of conducting and disseminating our research, healthcare professionals, members of the public and the academe often raised the question, ‘but is there truth in fiction?’. The question was not one we originally set out to answer, but we began to recognise it as an assumption embedded in our research, although cognisant of the complexity (Literature review). We were posed this question directly when presenting our project to diverse audiences, some baffled by what fiction might have to say about such an important health issue. Having carried out extensive empirical research on real readers’ responses to fiction about dementia, we now address the question by considering it theoretically (Literature review) and empirically (Analysis using reader response data). Our findings offer evidence for the depth and breadth of analysis that interdisciplinary research can bring to bear on complex health challenges such as dementia. They also offer insights into the relationship between fictional representations of medical conditions and their truth value in society, a key issue in the Medical Humanities.

### About the underpinning research and methodology

First, we provide a summary of the underpinning research project, ‘Dementia in the minds of characters and readers’. There is a rise in the reported diagnoses of dementia, as well as cultural representations of it, particularly in prose fiction. Narrative prose is a unique form, because it can grant access to the thoughts and internal perspective of a character. While some autobiographical dementia narratives exist, due to the progressive nature of the condition, those accounts do not describe the later stages, leaving fictional representations as the only ones available. These fictional texts allow readers access to an experience that is otherwise unavailable to them (Bitenc 2012; Bitenc 2020). Our research project aimed to:

1. Uncover the ways dementia is represented in the minds of fictional characters.
2. Explore how readers respond to extracts from the fiction, in order to understand the potential for fiction to represent a lived experience and perhaps facilitate understanding, awareness and/or empathy.

**Figure 1:**
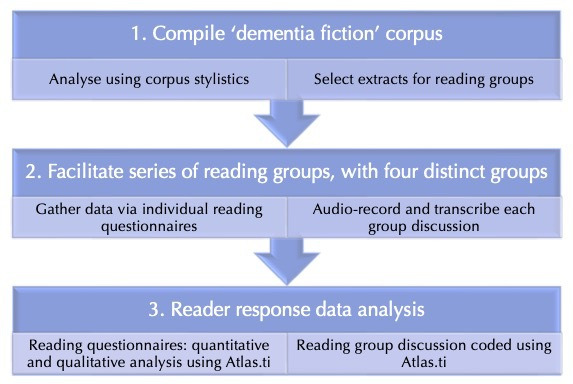
Research Design.

The project’s research design is summarised in Figure 1.

In Stage 1, Lugea gathered digitised texts of contemporary fiction from the perspective of characters with dementia, resulting in a 400 000-word corpus (see Lugea 2022a for a full account of the data selection process). Quantitative and qualitative analytical techniques from literary linguistics were applied to reveal the ways in which the experience of dementia is represented in fictional language (Lugea 2022a, 2022b). A snapshot of how literary language can simulate the experience of dementia is provided below. In Stage 2, the interdisciplinary research team selected extracts from the corpus. As well as choosing a range of scenarios, characters and contexts, we sought to include extracts rich in the textual features that represent the cognitive experience of the condition (as established by Lugea 2022a, 2022b). Further details of the extract selection process are outlined in Carney *et al* (2023). The extracts, numbered and summarised in table 1, were then used in a series of reading groups, in pursuance of aim 2 (above).

**Table 1:** Fictional extracts used in reading groups

Extract no.	Novel name	Author	Author’s relationship with dementia	Character name	Narrator type	Extract content
**1a**	*An Absent Mind*	Eric Rill	Novel inspired by author’s father w/ Alzheimer’s Disease	Saul	First-person	Saul’s family gather in his house to discuss his diagnosis. He recalls his tough guy days, showing he has a good long-term memory.
**1b**	*Elizabeth is Missing*	Emma Healey	Novel inspired by author’s grandmother w/ dementia	Maud	First-person	Maud breaks a vase in a department store and the shop assistant is unkind.
**2a**	*The Madonnas of Leningrad*	Debra Dean	Author has no personal or professional experience, but researched for creative writing.	Marina	Third-person	Marina forgets why she is in the kitchen if she has eaten. Her husband is affectionate and reminds her of the family wedding tomorrow.
**2b**	*The Madonnas of Leningrad*	Debra Dean		Marina	Third-person	Marina is getting ready for their trip for the wedding and her husband Dmitri helps her get dressed while she thinks about the past.
**3a**	*The Wilderness*	Samantha Harvey	Author has no personal or professional experience, but researched for creative writing.	Jake	Third-person	Jake remembers his fling, Joy, and thinks of himself through her eyes, then thinks of himself through his wife Helen’s eyes. These are two different versions of himself which he cannot reconcile with his own forgotten self-identity.
**3b**	*The Wilderness*	Samantha Harvey		Jake	Third-person	Under the advice of his doctor, Jake tries to create a timeline of his life events. He keeps deciding to make coffee and forgetting and takes comfort in his dog.
**4a**	*Still Alice*	Lisa Genova	Author is neuroscientist	Alice	Third-person	Alice is with her daughters, and although she has forgotten their names, she can enjoy quality time with them, her grandchildren and respond accurately to the emotion in Lydia’s rehearsed reading.
**4b**	*Turn of Mind*	Alice LaPlante	Novel inspired by author’s mother w/ dementia	Jennifer	First-person	Jennifer wakes to noises from other residents at night and tries to get up to prescribe them something. A carer with rough hands misunderstands and tries to take her to bathroom, disregards her request for coffee, calls her by her first name. She reflects on the meaning of care and shares a joke with a kind Filipino male carer.
**5a**	*Out of Mind*	Josef Bernlef	Unknown	Maarten	First-person	Maarten talks to his wife, Vera, and she tries to understand how he is feeling.
**5b**	*Out of Mind*	Josef Bernlef		Maarten	First-person	Maarten hears Vera describe his condition to someone in the kitchen and gets emotional. He thinks he is going to work and enters the kitchen, introduced to Phil, his new caregiver. She takes him for a walk.
**6a**	*The Things we Keep*	Sally Hepworth	Author has no personal or professional experience, but researched for creative writing.	Anna	First-person	Anna is sweltering in her room and tries to figure out why when her brother Jack comes to visit. Although she recognises him, she does not recognise Ethan, her favourite nephew, at first. He is upset, but then introduces himself and she tells him she loves him.
**6b**	*There But For The*	Ali Smith	Unknown	May	Third-person	May is in hospital, refusing to take pills and thinking about why she would not go into a residential home, recalling a visit there once.

A pilot study taught us that, in a group where people have a range of experiences of dementia, some participants felt they did not have ‘the same rights’ to speak (eg, a person with no lived experience compared their speaking rights to another person living with a diagnosis). Consequently, our main project comprised a series of reading groups with four distinct groups, listed in table 2.

**Table 2:** Reading group participants

Group name	Group abbreviation	No. of participants	Gender	Age	Recruited via
**Student social workers**	Students	9	6 female,3 male	7 (18–29), 2 (39–49)	Internal university social work undergraduate programme
**General public**	Public	9	8 female,1 male	18–69	Social media call, asking for people with little–no personal experience of dementia
**Carers**	Carers	6	4 female,2 male	40–79	Alzheimer’s Society carers’ network
**People with dementia**	Dementia	7	4 female,3 male	50–79	Dementia NI, an empowerment charity working with people with dementia in the community

The groups were not controlled for age or gender (further demographics are reported in Carney *et al* 2023). Rather, the four distinct groups reflect our project’s interest in understanding how a reader’s lived experience can affect their reading response. We sought the responses of people living with dementia for ethical reasons (Carney *et al* 2023) and to better understand how the fiction relates to their lived experiences.

The reading groups ran on a weekly basis for 6 weeks and adopted a ‘naturalistic’ approach—one that might feel like a book club—to capture readers’ sincere responses through ‘ecological validity’ (Hall 2008). As the COVID pandemic forced our sessions online, each session involved playing an audio recording of the extract being read aloud, after which the participants individually answered a questionnaire about their response to the extract, followed by a 15-min facilitated group discussion. This procedure was repeated using a second extract, except for the readers with dementia for whom the method was adapted for accessibility reasons, developed in partnership with Dementia NI, an empowerment charity (table 2).

The purpose of the individual questionnaire was to capture private, immediate responses and to mitigate the fact that group dynamics can shape responses gathered in interaction (Peplow 2016). The questionnaire for Groups A–C was based on cognate research (Fletcher and Monterosso 2016), adapted to address the project’s research aims regarding participants’ emotional and empathic responses in relation to past experience (or lack thereof) and the textual features (see Supplemental appendix 1). The questionnaire for participants with dementia was shorter and omitted questions exploring the effects of reading on them, in favour of questions about the relationship between the fictional extract and their lived experience (Supplemental appendix 1). In this way, we acknowledge that Group D was more primed than Groups A–C to reflect on the truth value of the fictional extracts. The project did not aim to investigate the ‘truth’ of the fictional representations, as the questionnaires indicate (see Supplemental appendices), yet the reader responses lend themselves to this purpose. While the questionnaires may have established the themes of the group discussions, the researchers emphasised the validity of any kind of response and made negligible contributions, save minimal prompts.

In this paper, we draw on the questionnaire data and reading group discussion transcripts, which were qualitatively coded using ATLAS.ti (Stage 3, Figure 1). Ethical approval was granted and participants are anonymised through pseudonyms. The research project inspired a significant range of outreach and engagement activities, as well as an anthology of new creative writing about dementia (Carson and Lugea 2022). Dealing with dementia fiction in all these ways—analysing the language, reading closely with people of different backgrounds, considering how it can be written well—provides the empirical and experiential evidence to address the question, ‘is there truth in fiction?’. For a full account of the project’s research design and methodology, see Devine *et al* (forthcoming).

### Fictional representations of dementia: an example

Telling a story involves using memory to verbalise a sequence of events, faculties that are challenged by dementia symptoms. Therefore, fictional narratives representing dementia involve a creative manipulation of information flow, temporal sequencing, spatial arrangements and viewpoints, all features present in our corpus and selected extracts. Furthermore, because narrative allows the representation of multiple perspectives and realities at once, it grants readers a wide-ranging view of the condition. Lugea (2022a) analyses the many ways in which contemporary fiction represents the subjective experience of dementia. For illustrative purposes, this section provides a brief stylistic analysis of a passage from an extract used in the reading groups (Table 1,2b).

*The Madonnas of Leningrad* (Dean 2006) tells the story of Marina, a Russian immigrant to the USA who lived through the siege of Leningrad by hiding in the vault of the city’s art museum. Although the novel is set while older Marina lives with dementia, there are frequent flashbacks to her younger years in Leningrad, emphasising the impact of her past memories on her current thoughts and feelings. Below is a passage from extract 2b, where Dmitri has just reminded his wife, Marina, that they are to attend their granddaughter’s wedding:

‘Yes, of course.’ She turns away from Dmitri and begins to fish around in her jewellery box. A wedding, so she should dress up. She will wear her mother’s… the things that hang from ears. She can picture them quite clearly but cannot find the word. Neither can she find the objects themselves. She could ask Dmitri where they have gotten to, but first, she needs the word. Her mother’s what? They are filigreed gold with little rubies. She can picture them, but there is no word with the picture, not in English or in Russian.

The narrator is ‘outside’ the story, using the third-person (‘She turns’), yet some features of the language are attributable to Marina and her symptoms. For instance, the question ‘Her mother’s what?’ is phrased as an interrogative, as if Marina were asking herself, even though it uses the third-person ‘her’. The use of indefinite article ‘a’ before ‘wedding’ indicates that the event is news to Marina, even though the event was established earlier in the story. Furthermore, the word ‘earrings’ is missing from the narrative, simulating Marina’s memory difficulties. These features correspond to Free Indirect Style (FIS), a narrative strategy that blends external third-person narration with features of a first-person viewpoint (Lugea 2022a). While the example above simulates the linguistic and cognitive difficulties in a character with dementia, the distance that the third-person grants also allows the writer to use language that the character might not be able to articulate in that moment, for example, ‘filigreed gold’. Although the words are attributed to Marina’s thoughts and long-term memory, the use of third-person means the external narrator is ultimately responsible for them. This allows the writer freedom of expression, as not all the words pertain to the ‘voice’ of the character with dementia.

First-person narratives, on the other hand, are fully attributable to the narrator with dementia (see table 1). Lugea (2022a) found that these dementia narratives often use the present tense, which helps to convey the salience of the present environment and sensory input when memory is affected. This section has provided a snapshot of how fictional narrative can represent the subjective experience of dementia, laying the contextual groundwork for the theoretical and empirical arguments to follow.

## Literature review

We now ask the question ‘is there truth in fiction?’ drawing on scholarship from a variety of disciplines. We begin by interrogating the significance of ‘truth’ in different domains, before outlining the issue from aesthetic and literary philosophical positions. Then, we draw on the Cognitive Sciences and empirical linguistics and literary studies to summarise some of the relevant cognitive processes involved in reading fiction, paving the way for the reader response analysis.

### Truth

Before exploring the capacity of fiction to carry truth, we might ask is there ‘truth’ at all. In our current post-truth era, increasingly populist societies are seeing ‘a rising tide of unconcern for truth’ (Haack 2019, 262) as well as distrust of institutionalised authorities, such as academic experts. Within the academe, truth is valued yet is a phenomenon difficult to pin down to varying extents in different disciplines. The Humanities and Social Sciences are generally more comfortable with the slipperiness or plurality of truth; in exploring humans, their languages, practices, cultures, what is ‘true’ is often relative or, as far back as Kant, understood as ‘constructed’. The hard sciences may sometimes pin down an ‘objective truth’ (eg, the sun sets and rises), but it is not always possible (eg, in medicine, Gillett 1995).

Yet—despite cynicism towards truth in the public and the search for truth in science—there must still be value in the concept. Whether or not it is methodologically attainable, truth is generally valued as a core principle of academic integrity. Outside of the academe, readers in our project responded to the dementia fiction by searching in it for some kind of truth (see analysis). As Haack observes, ‘There is one truth, but many truths; and while truth is objective, some truths are relative—they make sense only relativized to a society, a community, a theory […] etc’ (2019, 271). We argue that fiction—perhaps because of its fictional status—invites readers to consider its truth value, to explore the possibilities of human experience and interrogate issues relative to their subjective experience, community or society.

### Truth in fiction: theoretical approaches

The truth of fiction has been debated by scholars in literary theory, the philosophy of literature and aesthetics for decades (eg, see *Journal of Aesthetics and Art Criticism*: Clegg 1972; Hospers 1960; Mew 1973; Sparshott 1967; Mccormick 1983; New 1997). At the heart of the matter is the paradox inherent in ‘truth in fiction’: fiction, by its very nature, does not claim to tell the truth and, furthermore, it even uses forms that emphasise its fictionality and constructedness, such as metaphors, internal viewpoints and flashbacks (Riffaterre 1990); forms which are replete in our dementia fiction corpus (Lugea 2022a, 2022b) and contribute to the unique ways that fiction can present subjective experience in general and dementia in particular.

Lamarque and Olsen (1994) advance a ‘no-truth’ approach to fiction that became so influential it closed off the debate for some time (although see useful developments in Nielsen *et al* 2015 and Ryan 2019). Underpinning their argument is a narrow, Aristotelian definition of truth: ‘to say of what is that it is’. So when literature is said to correspond with the truth—to be like, or about the truth—they hold these looser correlations as invalid. Lamarque and Olsen critique existing scholarship for ‘the diffuseness of literary truth’ (1994, 11), listing five kinds of theories on the relationship between truth and fiction (1994, 12–14):

1. Mimetic (ie, fiction mirrors or represents reality that is, it is **accurate**).
2. Integrity (ie, fiction can be authentic or sincere; we call **authentic** below)
3. **Affective** (ie, fiction allows us to see as if, or feel as if; empathy)
4. **Epistemological** (ie, we can learn from fiction, gain knowledge about reality)
5. **Moral** (ie, ‘moral of the story’, fiction can teach us moral truths).

Like Pitari (2023), we disagree that the plurality of truths listed above is a reason to reject the relevance of truth to fiction altogether. We adopt a more diffuse definition of truth, one that includes these five ways that fiction may correspond with reality, in order to facilitate a scholarly investigation of the possibilities. Lamarque and Olsen’s ‘no-truth’ approach ignores (1) the fact that lay readers are interested in how fiction corresponds with lived experience, and (2) that the value of literature—which they are keen to emphasise—rests on its capacity to offer some kind of truth about the real world. The following subsections take Lamarque and Olsen’s list of five theories as a departure point for discussing the kinds of truths that fiction can engender and we do so with reference to empirical evidence.

#### Mimetic theories: fiction can be accurate

Mimetic theories understand fiction as mirroring reality, to varying degrees and measures of accuracy. Fiction can be considered as a narrative representation of imagined events. Considering texts as ‘representations’ entails recognising their constructedness. Texts, as constructed representations, are shaped by the context(s) of their production and reception, including prevailing attitudes, discourse practices and ideologies. In our study, the corpus of dementia fiction was drawn from the last 35 years, acknowledging that contemporary representations of the condition would differ from older ones. We focused on dementia fiction in the English language; despite searching for fiction written by or featuring a range of people, we were struck by the lack of diversity in terms of class and ethnicity. Therefore, we acknowledge that our corpus and selected extracts were particular kinds of representation (Anglophone, generally white Western and middle class), representing particular kinds of ‘truths’ about dementia (see table 1 and, for further details, Lugea (2022a) on the corpus and Carney *et al* (2023) on extract selection). Our anthology of commissioned stories aimed to redress the lack of diversity (Carson and Lugea 2022).

By focusing on a particular experience, any given story is a selective representation of the truth. One kind of accuracy may be prioritised over another, but the medical accuracy of illness narratives is always significant. Based on her research on fictional representations of autism, Semino (2014, 143) remarks,

‘[…]the fictional representation of autism may not be, or aim to be, accurate from a clinical point of view. Nonetheless, while medical accuracy is not required for the success of a fictional narrative, stories involving characters with illnesses or disorders tend to be valued, among other things, for their degree of realism. This requires consistency, at least to some extent, with general perceptions of those conditions.’

Thus, she suggests that medical accuracy may not be necessary but ‘some degree of realism’, even coherence with ‘general perceptions’, can lend value to fictional representations of medical conditions. Sometimes, illnesses in fiction are medically inaccurate: for example, a text not included in our study is New York Times #1 bestseller *The Vanishing Half* (Bennett 2020) which overplays the role of genetics in Alzheimer’s Disease, perpetuating an inaccurate myth.

Nevertheless, medical discourse can be incorrect or partial and should not be considered the sole arbiter of mimetic truth. In our corpus, the neuroscientist Lisa Genova’s novel *Still Alice* is medically accurate. Some critics point out that *Still Alice* perpetuates stereotypes of dementia, with regards to the ‘narrative of decline’ (Falcus 2014) and the idea that selfhood is based on communicative ability (Bitenc 2020,71). Under correspondence theory, such stereotypes are not necessarily falsehoods, yet the example underscores the selectivity and social constructedness of all fictional representations. The point is that accuracy is not just medical, but also social and cultural, and fictional representations have the capacity to challenge or confirm socioculturally held prejudices. Carson (2022) emphasises the responsibility of authors to conduct careful research when depicting a lived condition in their writing, while upholding the right to creative license. That creative license may be uninformed, or informed by genre; see Ryan (1991a, 1991b) for a taxonomy of ways that fictional genres (such as speculative or science fiction) depart from real-world truths.

#### Integrity theories: fiction can be authentic

Approaches to fiction that evaluate the extent to which it authentically represents lived experience are called ‘integrity theories’ by Lamarque and Olsen. The social semiotician, Van Leeuwen, describes **authenticity** as ‘concerned more with the moral or artistic authority of the representation than with its truth or reality’ (2001, 397). Therefore, evaluating fiction as authentic involves a judgement on the part of the reader as to the validity of the author’s representation.^2^ In her study of realism and authenticity in war narratives, Nuttall finds that readers believe authors ‘must have ‘legitimacy through experience’ or have actually witnessed the events for themselves’ (2019, 217). The dementia fiction we studied was written by people without a dementia diagnosis (for reasons outlined earlier) so their experience could only be external. Some of the authors have indirect experience of dementia in a family member or, alternatively, their authority is earned through research, scientific or otherwise (table 1). Our inclusion of readers living with dementia in the research served to evaluate the authenticity of the fictional representations and, as reported in our analysis, this group widely endorsed the extracts read.

Even though it is impossible to access other peoples’ minds, fiction can construct and grant access to another person’s reality, which Nuttall (2019) argues creates an impression of realism for the reader. This impression of realism is forged through narrative forms and strategies that, on the one hand, emphasise fictionality but, on the other, construct something that can feel real (literature review). Nuttall emphasises that realism and authenticity are ‘perceived qualities of narrative’ and that readers’ judgements are very much dependent on ‘the specific contexts of production and reception for individual texts’ (Nuttall 2019, 232). We acknowledge that the institutional authority wielded by academics may have resulted in, our reading group participants assuming the fictional representations were ‘true’. Nonetheless, our method allowed readers opportunities to explore and challenge the relative truth of the fictional extracts, as their responses in the analysis show.

#### Affective theories: fiction can make us feel

Fiction corresponds to truth by allowing readers to vicariously live through another person’s truth, to see or feel as if they were ‘in their shoes’; earlier, we highlighted some of the stylistic features of fictional narratives that facilitate this perspective-taking. Research indicates that readers’ engagement with narrative can evoke ‘narrative empathy’ (Keen 2006, 2007). Furthermore, because fiction evokes **affective** responses towards imagined people and situations, it provides ‘safe zones’ for readers to discuss challenging issues such as dementia. As Keen notes, ‘fictionality offers a no-strings attached opportunity’ (Keen 2007, 168) for readers to engage with characters away from any ‘demands on real-world action’(Keen 2007, 4). So affective truth allows a profound level of emotional engagement with a real-world issue without any real demands on the reader. Our reading group participants confirmed that fiction provided a useful distance, as well as a powerful level of engagement, to explore an important health and social issue. The feelings that arise from reading fiction—which can be strong (see analysis)—can then affect our real-world understandings, attitudes and behaviours, fiction’s ‘epistemological and moral truth’, as discussed in the next section. Our findings with regards readers’ emotional and empathic responses are reported elsewhere (Fernandez-Quintanilla *et a*l, forthcoming).

#### Epistemological and moral theories: fiction can teach us things

Fiction can impart a truth that enhances our understanding of reality (**epistemological** truth) or our sense of right and wrong (**moral** truth). Although Lamarque and Olsen (1994) distinguish these theories, we approach them together because both are about lessons we can take from fiction to the real world. The emotive and empathic effects of fiction are such that they can provoke real-world changes in our understanding, for which we have empirical evidence (Fernandez-Quintanilla *et al*, forthcoming). The same questionnaire was given to participants in Groups A–C before and after the series of reading groups, to assess the impact of participation in the reading groups. These participants reported increased levels of awareness and understanding towards dementia. Furthermore, over half the participants in Groups A–C reported that the reading groups impacted on their behaviour in situations involving dementia. While our study did not follow the participants’ subsequent actions, some research has explored the link between reading fiction and prosocial behaviour, for example, donating to charity, volunteering (Kidd and Castano 2013; Koopman 2015). Fictional narratives can shape public discourse, having real-life implications for public attitudes, understanding and debates (Nuttall 2019; Semino 2014), as well as policy-making and care provision (see below, Bringing a character to life).

### Reading fiction: blending text and minds

So far we have summarised approaches to truth in fiction from literary theory and philosophy; now, we turn to relevant scholarship in Cognitive Stylistics, which uses insights from the Cognitive Sciences to explore readers’ processing of literary texts (Lugea and Walker 2023; Semino and Culpeper 2002; Stockwell 2020). According to Cognitive Psychology, information is held in our minds in packages, known as ‘schemata’ (Schank 1986). Schematic knowledge is forged through individual experiences, as well as culturally and socially conditioned. When we read, we create mental impressions of texts by blending incoming textual information (‘bottom-up cues’) with schematic knowledge (‘top-down cues’). This blend of processes accounts for how readers produce different interpretations of a given text, as they bring different sets of experiences and expectations to bear.

Likewise, a combination of bottom–up and top–down cues creates impressions of characters in readers’ minds (Culpeper 2001; Culpeper and Fernandez Quintanilla 2017). The text provides descriptions of physique, movement, dress, gesture and behaviour—linguistic and otherwise—from which character can be inferred. We tend to ‘interpret fictional characters, despite their imaginary status, in large part with knowledge about people acquired through our real life experiences’ (Culpeper and Fernandez Quintanilla 2017, 95), a claim for which there is empirical evidence (Gernsbacher, Goldsmith, and Robertson 1992; Graesser, Singer, and Trabasso 1994) and that our own evidence corroborates (eg example *v*, below). Together, textual information and our schematic knowledge about people create characters that are purportedly ‘richer than the denoted information provided by the text’ (Stockwell and Mahlberg 2015, 134). Somehow, the sum is more than the parts, as characters come to life in readers’ minds. The effect can be so powerful that fiction and its characters may then inform our real lives; for instance, by evoking an empathic response to similar people in real contexts, or moving readers to prosocial behaviour. The interplay between a text and a reader’s knowledge and experience, summarised here, is relevant to understanding truth in fiction because fiction is read—and characters constructed—by relating textual information to what readers ‘know’ about the real world and its people; that is, readers’ perceptions of what is true.

## Analysis using reader response data

In the analysis, we draw on questionnaire and discussion data gathered through the reading groups and coded in ATLAS.ti by three researchers. This analysis draws on the codes that are most relevant to the question—is there truth in fiction?—which are also some of the most widely applied codes in our data. Table 3 lists the relevant codes, their definitions and groundedness scores that is, the number of quotations from the data coded with that code (Gr). The final column also indicates the groundedness score for each participant group. Group D read half the extracts so have lower groundedness scores. Where relevant, the discussion refers to the number of co-occurrences between a pair of codes (n.), which indicates that the two concepts co-occur in the readers’ responses. We also searched ATLAS.ti for the words ‘true’ and ‘truth’ and synonyms, extracting relevant results for discussion below.^3^

**Table 3:** Relevant ATLAS.ti codes from qualitative reading group data

Code definition	Code name	Groundedness score, Gr*	Breakdown of groundedness score†
**Person with dementia refers to the book being ‘too realistic’ or ‘too heavy’.**	*#Story too close to the bone for pwd*	8	In relation to extract 2a(7),to 6a(1)
**Reader explicitly validates the authenticity of the character’s experience, either relating it to a specific incidence in real life, or mentioning that it was ‘interesting’ or similar**.	*#validates authenticity of extract*	128	Group A(6),B(5),C(53),D(55)
**Readers attribute feelings to the character with dementia, whether made explicit in the extract, or imagined**.	*ATT Feeling to character w/dementia*	480	Group A(180),B(144),C(89),D(32)
**Readers attribute speech or thought to the character may be feeling, even if it is not explicitly stated in the extract**.	*ATT speech/thought*	119	Group A(59), B(44), C(14),D(2)
**When participants observe that the character with dementia is more than their illness that is, observe their abilities, skills, traits, etc, regardless of dementia**.	*CHAR ATT - personhood*	99	Group A(42), B(40), C(11), D(6)
**When the reader compares themselves with:** **Caring character** **The character with dementia**	*COMP w/self:* *caring character* *character with dementia*	Total Gr. 152 50 102	Group A(7), B(4), C(39) D(0)Group A(20) B(22), C(9), D(51)
**When the reader compares a real person (not self) to character with dementia**	*COMP w/ real person: Char w/ dementia*	269	Group A(38), B(38), C(148), D(3)
**When the reader evokes some aspect of their own lived experience in interpreting the extract, with all the subcodes indicating different kinds of experience**.	*LIV EXPERIENCE:* *Carers feeling* *Clash with experience* *Family dementia* *Family conflict* *Family/carer perspective* *Indirect dementia* *Living with dementia* *Loss of activity after illness* *Other*	Total Gr. 47333413991763776556	Group A(60),B(61),C(249),D(103)Group A (2), B(1), C(30), D(0)Group A(0), B(0), C(39), D(2)Group A(15), B(11), C(13), D (0)Group A(0), B(0), C(2), D(7)Group A(17), B(8), C(141), D(10)Group A(14), B(13), C(10), D(0)Group D(76)Group C(1), D(4)Group A(11), B(28), C(13), D(4)
**Reader states how the text takes them back to the very moment; that is, there’s some form of enactment of the experience**	*READ_relive experience:*	42	Group A(1), B(0), C(21), D(4)
**Reader openly questions whether the fictional account of living with dementia is valid according to real life experience**	*RESPONSE_questions fictional account:*	83	Group A(5), B(11), C(55), D(12)
**Reader discusses the use of metaphor in the extract**	*STYLE_metaphor*	Total Gr. 117	

* In ATLAS.ti groundedness score (Gr.) is the number of quotations coded to that code.

† Group A=student social workers, Group B=general public, Group C=carers, Group D=people with dementia. Group D read only 6 of the 12 extracts so report smaller groundedness scores.

### A poetic voice: (in)authentic?

First, we consider the instances where our readers objected to the fictional representations, which were rare but revealing. Overall, very few participants answered ‘yes’ in response to Q20 ‘Did the extract contradict your experience of dementia?’ (21/257), indicating a general correspondence between the fiction and participants’ experience and expectations that is, a **mimetic** truth. When that minority were prompted to explain how the extract contradicted their experience, the majority of respondents were carers (14/21), who believed that the characters with dementia displayed too much detail in their awareness or memory. Likewise, in the group discussion, when readers questioned the truthfulness of the fictional account of dementia, the code *RESPONSE_questions fictional account* was applied (table 3). Again, the vast majority of these doubts were raised by Group C in relation to five extracts: 2a, 2b, 3a, 3b and 6b. As table 1 shows, these extracts are all from third-person narratives; specifically, ones that used Free Indirect Style. In the following quote, a carer questions the language used in extract 3a:

*i.* ‘I just don’t think that those words would be available to somebody […] with Alzheimer’s dementia. I’m not sure with other dementias, but you know, that is poetic license’ (Mary, Group C, on 3a)

Mary recognises that the author is using ‘poetic license’ but the attribution of poetic and expressive language to a character with dementia jars with her experience of her husband’s Alzheimer’s disease. It seems the author’s use of FIS to blend the words of the narrator and the character result in a voice that, for the carers, is too unlike what they observe in their loved ones. The fact that only the carers expressed these misgivings indicates that lived experience strongly conditions readers’ evaluations of the **accuracy** and **authenticity** of fictional representations.

Overall, the carers endorsed the truthfulness of narratives that attributed simpler language to the characters with dementia, including all the first-person narratives, as well as the third-person narrative *Still Alice*, which uses very little FIS and instead employs increasingly short, simple sentences in the present tense. In response to an extract from *Still Alice*, Jemima says:

*ii.* ‘And I just think it is a powerful message. If somebody was reading that who hadn’t had the experience that we have with people with dementia […] I think that would really push it across to them that […] they do feel it, even if they can’t express it […]. And I honestly think this is the best […] as far as being true to how it is, you know, as true as you can be.’ (Jemima, Group C, on 4a)

Based on her caring experience, Jemima agrees with the extract’s message: that people with dementia have feelings, even if inexpressible. This strong response from Jemima helps explain how the carers’ experience is privileged insofar as they are painfully aware of the existence of underlying feelings in their loved ones, but also restrictive in that the ‘voicing’ of those thoughts and feelings in fictional narrative appears inauthentic.

It is interesting to note that readers living with dementia (Group D), never questioned the same extracts in the same way. For them, the representation of expressive inner thoughts and detailed memories in characters with dementia was unremarkable. The distinction here may be explained by the fact that carers only have access to the external presentation of dementia in their loved ones, not their inner lives. For those living with dementia, the expression of memories, thoughts and feelings in poetic language and detail was not a barrier to its accuracy; in fact, as the next section shows, they felt it helped express the truth they are living through, providing evidence for the **epistemological truth** in fiction; that is, that fiction can teach us something otherwise inaccessible in life.

### Fiction as ‘the whole truth’

The stylistic analysis provided earlier illustrated how narrative fiction can blend perspectives (eg, through FIS), traverse time and space, and represent alternative ways of thinking. Although some aspects of this style were too expressive for the carers, other groups found it granted an enhanced perspective. In response to extract 2a, a reader with dementia attests:

*iii.* ‘[I]t is extremely true to life. My father had Alzheimer’s so I seen what was going on with him […]. The author of this book is incredibly in control of the story. Just that wee, short extract pulls you right into the book, the whole story taking you into Leningrad and the museum and exporting you to America, so […] you have the whole concept of what’s going on all around you. Now for this book to be fictional, it’s very, very well-researched. I mean, it could well be an absolute person’s truth, you know, a whole life.’ (Anthony, Group D, on 2a)

Anthony’s response was coded *#validates authenticity of extract*. Anthony has lived and observed experience of dementia. He praises the author for her research, judging the fictional representation as **authentic**. He evaluates it as ‘true to life’ but emphasises the techniques that the author uses to convey ‘the whole concept’; for instance, he observes how the narrative provides multiple perspectives, across time and place, including the character’s past and present. He may also be referring to the fact that the narrative uses the third person, observing Marina from outside, but also gives access to the workings of Marina’s mind. As FIS, this is the kind of narrativisation to which the carers objected, yet here Anthony observes how fiction can capture multiple truths at once: internal/external, past and present. This reader’s remarks lend empirical support, from the perspective of a person living with dementia, for the ways that fictional language lets readers both simulate the condition and understand the person behind the condition, who they are and who they were (Lugea 2022a). The techniques used in fictional language may be artifice, but they simulate a truth that corresponds with reality.

### The power of metaphor

Despite some misgivings, the carers were very moved by the literary representations of dementia. Perhaps inevitably given their life experience, they reported higher levels of emotional intensity than other groups; When asked ‘Q12. How strong were the emotions you felt during reading?’, in almost two-thirds of their responses, Group C selected ‘significant’ or ‘very strong’, significantly more than Groups A and B.^4^ Furthermore, their responses often positively evaluate the use of literary language to represent the condition’s symptoms, as Matt does:

*iv.* ‘One section, I thought was a just beautiful bit of writing; ‘Like a ship, a sailing vessel that is becalmed. And then suddenly, there is a breeze. I’m sailing again. Then the world has a hold on me again’. I thought that sums it up perfectly.’ (Matt, Group C, on 5a)

Matt is struck by the metaphors which describe the protagonist feeling like a ‘ship’, at the mercy of the elements, moved and calmed by external forces. The use of metaphor is often hailed as a hallmark of literature and is arguably one of the signs of its fictionality (Riffaterre 1990); that is, a person is not a ‘ship’ and to say so is untrue. But to apply the concept of a ship on to a person in the early symptoms of dementia is a rhetorical device that gives the reader a way of conceptualising the experience. Research shows that metaphors can be useful to describe abstract or subjective experiences (eg, pain, cancer) in more concrete terms (Neilson 2016; Semino *et al* 2020). The ‘ship’ metaphor invites readers to bring personal knowledge of ‘being at sea’ to bear in imagining how the character is feeling. Matt has an aesthetic response to the passage (‘beautiful bit of writing’) but also ascribes the literary representation with a **mimetic** truth value: ‘sums it up perfectly’.

Matt’s quote typifies what we found in the qualitative analysis of all the groups’ discussions. When participants discussed the metaphors, this was coded (*STYLE_metaphor*, Gr. 117). ATLAS.ti analysis reveals which codes co-occur, indicating relationships between concepts. In the 117 instances where participants’ quotations were coded for discussing metaphor, there were 69 co-occurrences with the code *ATT feeling to character w/ dementia* (see table 3), indicating that metaphors help readers understand characters’ emotional states. Readers also discuss metaphors when professing that they have gained insight into the perspective of the character with dementia (34 co-occurrences), when expressing an emotional response to the text (28 co-occurrences), or positively evaluating the text (26 co-occurrences). These findings suggest that literary metaphors evoke emotional responses and positive evaluations of the extracts and may help readers’ understand the experience of dementia.

### Fiction as a ‘real sense’

Thus far, we have presented evidence from the readers’ responses to support the idea that fictional representations, despite or because of their artifice, can feel ‘real’. When we searched for ‘truth’ and synonyms across the readers’ responses, we came across several uses of the idiomatic phrase ‘a real sense’, limited to Groups A and B. The phrase is notable for this paper because it implies that a sense or impression arising from a text can feel real but—importantly—that it is not real.^5^ The phrase supports the **mimetic** theory of fiction as simulating reality. The following quote exemplifies its use; here, a member of Group B agrees with her fellow participants:

*v.* ‘I liked it as well. And I think it was possibly because you can see some people in it. Like, I’ve known people who have hidden their pills or not wanted to do something. So, I got a real sense that she was very determined and no-nonsense, even though she was in a very physically vulnerable state.’ (Lorraine, Group B, on 6b)

Lorraine’s ‘real sense’ of the character, May, is formed by two things: first, the textual description of May and her behaviour and, second, Lorraine’s experience of real people with similar behaviours. Her account provides further evidence for the theory that impressions of characters are formed when readers combine bottom–up and top–down cues. This example illustrates how the text and real-world knowledge about people are processed together to from an impression of fictional characters: Lorraine concludes from both evidence sources that May is ‘very determined and no-nonsense’. The character’s behaviour and Lorraine’s real-world experience are not necessarily dementia-related, but serve to provide a realistic depiction of this character, to whom Lorraine responds favourably.

The fact that the phrase ‘real sense’ occurs only among the participants in Groups A and B indicates how readers’ lived experiences inform their impressions of a fictional text. Groups A and B have less experience of dementia, so their impressions are largely text-driven, using bottom–up cues from the text. Having a ‘real sense’ is limited to those readers whose ‘sense’ is stimulated by the text which, despite being fictional, has a realism to it. On the other hand, Groups C and D have in-depth experience of dementia, so their impressions are largely informed by what they already know. It seems this phrase is redundant to those with lived experience. In contrast, Groups C and D were more likely to pronounce the extracts as ‘authentic’ (table 3), which indicates that their lived experience of dementia grants them the authority to do so and that it was reflected in the fiction.

### Bringing a character to life

Example v, above, illustrates how readers attribute personality to characters, based on textual cues and real-world knowledge about people. Table 3 data also shows how readers attribute speech and thoughts to characters (*ATT_speech/thought*, Gr.119), as well as hypothesise about how they are feeling (*ATT_feeling*, Gr. 480). These two codes, among the highest groundedness scores, are significant because they involve readers treating characters as if they were real, to the extent that they can put words in their mouths and attribute them with feelings. These codes are most frequently applied to responses from Groups A and B because, with their limited experience of dementia, they rely on ‘bottom-up’ textual cues to form impressions of characters.

In the following quote, Timothy, a student social worker, discusses extract 5a in which the character, Maarten, is becoming aware of his dementia symptoms and his inner thoughts are presented in a stark contrast to his outward appearance and interaction with his wife, Vera.

*vi.* “Just looking at how Vera came across in this excerpt. I found her quite endearing in a way. Because, and this is my social worker training trying to maybe poke its head through, in learning about empathy… So, if I imagined myself speaking to Maarten, for example, it wouldn’t be very helpful to pretend that I know what it’s like, or to try to put a silver lining on things that maybe he doesn’t want to hear. But the way Vera comes across is genuine congruence and admitting that ‘look, you know, I find it hard to—I can’t understand what this is like for you’. And she seems to be genuinely trying to, you know, listening to his explanations, and not feigning that, ‘Oh, yeah, I understand, you know, I know all about dementia’ and I don't think that’d be very helpful. So, I thought Vera set a good example, for the kind of attitude to maybe adopt when you’re speaking with or working with people with dementia.’ (Timothy, Group A, on 5a)

The ‘quotes’ attributed to Vera are not actually in the fictional extract, but are Timothy’s hypotheses about what the character is saying or, even, not saying. As a student social worker, Timothy focuses on Vera and Maarten’s interaction and ‘imagines’ himself interacting with a person with dementia. The extract lends Timothy an understanding of the contrast between Maarten’s internal perspective and outward behaviour. Timothy—and readers in general—can gain insight into the multiplicity of perspectives on one situation because fiction can present and contrast different perspectives, that is, truths relative to different people. When the character Vera acknowledges the distinctions in their perspectives, the story content coheres with the story’s form, further validating this as a potentially useful approach in social work, facilitating a more active and open ‘listening’ or ‘empathy’. By granting multiple viewpoints at once, the fiction lends an enhanced understanding, one that allows this reader to ‘imagine’ both perspectives as valid and elaborate a useful interactive style to bridge the communicative gap.

A member of the general public, Group B, had a similar response to this extract: ‘her attempts at understanding were so genuine that you could feel something of what it was like for Vera.’ (Nora, Group B, on extract 5a). Both Nora and Timothy use the word ‘genuine/ly’ to describe Vera’s approach in 5a, indicating that they view her intentions as honest and even her character as real. Their responses evidence how readers humanise fictional characters and support the theories that fiction can mirror and authentically represent reality. Furthermore, Nora ‘feels’ the fictional character’s experience, supporting the **affective** theories of fictional truth, and Timothy learns from the fiction, supporting the **epistemological** theories. As these examples are typical of our reader response data more generally, we suggest that a reading of fiction as ‘mimetic’ or ‘authentic’ facilitates an affective or epistemological response. That is, when readers validate a fictional representation as accurately or authentically representing a reality, an empathic or enlightened response is more available to them. Furthermore, our project provides evidence for the capacity for fiction to facilitate understanding and empathy of lived experiences (Fernandez-Quintanilla *et al*, forthcoming), acknowledging the complexities around the concept of empathy in the Medical Humanities (Whitehead 2017) and beyond.

### Recognising a character as a person

We have just demonstrated how readers responded to fictional characters as if they were real, attributing words, thoughts and feelings to them. This section explores how the readers recognised the characters as a whole person, more than just a conglomeration of dementia symptoms. This is important because, in Dementia Studies, it has long been observed that society tends to view those with dementia as stripped of their ‘personhood’ (Kitwood 1997); this is attributed to the potential impact of dementia on an individual’s memory and communicative abilities, faculties which are often held as essential to ‘being a person’. When participants noted that the character with dementia is more than their illness (ie, observed their abilities, skills, traits) we applied the code *CHAR_ATT_personhood* (see table 3). Compounding our findings that those without lived experience of dementia form impressions based on the text, table 3 shows that the code was mostly applied to responses from Groups A (students) and B (general public), indicating that the fiction conveys to them a character who is ‘more than’ their diagnosis, something they may not have considered before. This finding indicates the increased awareness fiction can facilitate about real people and their lived experience that is, an **epistemological** truth.

To varying extents, the characters we studied had characteristics and lives beyond their diagnosis. In extract 6b, for instance, the character May is humorous, suspicious and defiant towards caregivers. One member of Group B (general public) responded to May’s character with delight:

*vii.* ‘[with dementia] you’re still the person you are and sometimes we need to remember that. I loved her personality. I love the fact she’s fighting back […] the writer is an imaginative person, they’re speculating as to what it might feel like […] and presumably she has observed people who may be close to her who have dementia. So, she’s kind of working from some experience. I loved it. I thought it was also very respectful of the person in a lovely—you know, people can be very reverential. All this ‘sorry for you’, […] and reverential is not a good human interaction with the person. Be real, be […] sad with them or for them, or be happy with them or just enjoy their personality and the person they are. And I think that’s a great message from any text. I loved this.’ (Ann, Group B, on 6b)

Ann assumes that the writer’s imaginings are based on real observations: upholding the ‘legitimacy through experience’ dogma by viewing truth as based on **authenticity**. Moreover, Ann positively evaluates this extract because of the ‘personality’ of the character with dementia, who fights back against the stereotypical loss of agency associated with dementia (van der Byl Williams and Zeilig 2023). The fact that the reader is able to infer a personality indicates that the brief extract conveys an **accurate** depiction of a person. She interprets ‘the message’ of the extract as one about the importance of being ‘respectful’ towards those with dementia, but not ‘reverential’. She dismisses reverential treatment in favour of interactions that spark human emotion, which she values for being ‘real’. As such, she imbues the fictional representation with **epistemological** truth—it reflects the reality that ‘with dementia you’re still who you are’—as well as with **moral** truth—that we should meet those with dementia on an equal footing. In doing so, this reader implicitly questions the **affective** dimension (‘All this ‘sorry for you’’); in this way, Ann chimes with more recent discussions of empathy within the Medical Humanities which question its perceived inherent value, especially when it may engender power asymmetries or block the potential for other kinds of interactions and socio-political change (Whitehead 2017).

## Conclusion

This article has explored the question—is there truth in fiction?—with recourse to a wide range of disciplinary perspectives, as well as a dataset of reader responses to dementia fiction. Our approach has been based on the correspondence theory of truth; that is, that truth corresponds to reality in certain ways. We have empirically investigated the ways that dementia fiction can correspond to readers’ truths, drawing on the five different kinds of correspondences outlined by Lamarque and Olsen: accuracy, authentic, affective, epistemological and moral. Our findings show that accuracy and authenticity are key to readers’ impressions of truth in fiction and suggest that they create the conditions for readers to establish affective, epistemological and moral truths. That is, once readers identify a fictional text as having an accurate and authentic truth value, they are more likely to respond with feeling, empathy and a change in their understanding or values. Insights from cognitive approaches to fictional reading have informed our analyses of how the reading group participants interpret the fictional extracts, as well as the characters and experiences depicted therein. Our findings lend empirical support to the idea that readers’ impressions are informed by combining ‘top–down’ and ‘bottom–up’ processing. The findings also indicate that the extent to which a reader has lived experience of a condition depicted in fiction dictates the extent to which they either draw on that experience, or rely on the textual cues to form their impression. Therefore, fiction has an ongoing role in the interrogation and establishment of the ‘truth’.

Our reader response data is subject to further analysis regarding these responses (Fernandez-Quintanilla *et al*, forthcoming). In the meantime, the present contribution goes some way to addressing the question—is there truth in fiction?—with, for the first time, empirical evidence drawn from a corpus of responses from different kinds of readers. We hope the enhanced understanding of truth in fiction, as offered here, contributes towards the Medical Humanities debate on the relevance of fictional representations of an illness to sociocultural understandings of that illness. Whether or not empathy is an achievable (or desirable) outcome of reading fiction about an illness, our findings have shown that fiction—because of and through its fictional status and form—provides readers with a means to interrogate the truth.

## supplementary material

10.1136/medhum-2024-012976 Supplementary file 1

## Data Availability

Data may be obtained from a third party and are not publicly available.

## Footnotes

For the purposes of this article, ‘fiction’ is understood as a series of imaginative statements, ‘reality’ is an objective state- of- affairs and ‘truth’ is a statement whichthat corresponds to reality. This definition of ‘truth’, known as the Correspondence Theory, is the most widely- held in Philosophy (Bourget and Chalmers 2014).

‘Moral’ authority is used here (by Van Leeuwen and us) in the classical rhetorical sense of a speaker’s *ethos* or moral authority, rather than in the more general sense of moral right/wrong (Section 2.2.4).

Examples of irrelevant results are the use of the word ‘truth’ or synonym by a researcher rather than a participant, or an idiomatic use of the word for example, ‘to tell the truth’.

In response to the same question, the responses ‘significant’ or ‘very strong’ were selected by Group A in just over one -quarter of their responses and by Group B in almost half of their responses. Group D (people living with dementia) were not presented with this question (Section 1.2).

Further evidence for our interpretation of ‘a real sense’ is found in the British National Corpus (BNC), a database of British English texts, carefully compiled by linguists to represent contemporary British English. The corpus reveals that the phrase occurs most frequently (per million words) in written texts about the ‘Arts’ (1.67) and ‘Belief and thought’ (1.98), indicating that the phrase is used to interpret meanings and explain interpretations.
