# Supplementary material for: Is there truth in fiction? Lessons from readers’ responses to dementia fiction
Source: Med Humanit. 2024 Dec 4;51(1):e012976. doi: 10.1136/medhum-2024-012976 (PMC11877083; doi:10.1136/medhum-2024-012976)
Supplement: Supplementary file 1 [file medhum-51-1-s001.pdf]

Appendix 1. Example Reading Questionnaire for Groups A, B and C

Please complete this questionnaire after reading - and marking-up - extract 1b from *Elizabeth is Missing*.

1. Looking at the markings you made on the extract, how and why did those bits strike you? ______________________

2. Have you read this book, *Elizabeth is Missing*, before?
- ☐ Yes
- ☐ No

3. Do you have personal experience of anything in this extract?
- ☐ Yes
- ☐ No

4. If yes, can you explain? ______________________

5. Did you feel like the extract was presented through a particular character's eyes?
- ☐ Yes
- ☐ No

6. If yes, whose eyes?
- ☐ Maud
- ☐ The shop assistant
- ☐ Other

7. If you selected 'other', whose eyes do you think the extract is presented through?

____

8. To what extent could you understand Maud's point of view?
- ☐ Not at all
- ☐ Slightly
- ☐ Moderately
- ☐ Considerably
- ☐ Completely

9. What was it about the extract that made you understand Maud's viewpoint?

____

10. To what extent could you understand the shop assistant's point of view?
- ☐ Not at all
- ☐ Slightly
- ☐ Moderately
- ☐ Considerably
- ☐ Completely

11. What was it about the extract that made you understand the shop assistant's viewpoint?

____

12. How strong were the emotion(s) you felt during reading?

- ☐ None
- ☐ Mild
- ☐ Moderate
- ☐ Significant
- ☐ Very strong

13. Can you describe the emotion(s) you felt while reading? If your emotion(s) relate to a specific moment in the extract, please specify.

____

14. If the emotions you felt relate to a specific character, please specify:

____

15. How do you think Maud, the character with dementia, was feeling?

____

16. To what degree did you feel the same emotions as Maud?
- ☐ Not at all
- ☐ Slightly
- ☐ Moderately
- ☐ Considerably
- ☐ Completely

17. If you felt a different emotion, can you name it: ______________________

18. Did reading this extract make you think about the experience of living with dementia in a new or different way?
- ☐ Yes
- ☐ No

19. If you answered *yes*, please explain: ______________________

20. Was there anything in this extract that contradicts your experience of dementia?
- ☐ Yes
- ☐ No
- ☐ Not applicable

21. If you answered *yes*, please explain: ______________________

22. Any other comments on this extract? You can use this space to provide responses that you may not want to give in the group discussion. ______________________

23. Please enter your first name and surname: ______________________

24. Please select the reading group you are participating in:
- ☐ A. QUB Social Work students
- ☐ B. General Public
- ☐ C. Carers

Appendix 2. Example Reading Questionnaire for Group D

Session 1. Reading Questionnaire
This questionnaire is to be completed during our reading group meeting. We will do it after reading the extract from 'An Absent Mind'.

1. If you made markings on the extract, how and why did those bits strike you?

2. This extract is from the book 'An Absent Mind'. Have you read this book before?
   Yes ☐
   No ☐

3. Do you have personal experience of anything in this extract?
   Yes ☐
   No ☐

4. If yes, can you explain?

5. Did reading this extract make you think about the experience of living with dementia in a new or different way?
   Yes ☐
   No ☐

6. If you answered yes, please explain:

7. Was there anything in this extract that contradicts your experience of dementia?
   Yes ☐
   No ☐

8. If you answered yes, please explain:

9. Any other comments on this extract? You can use this space to provide responses that you may not want to give in the group discussion.

10. Please enter your full name:

____
